# In-line balanced detection stimulated Raman scattering microscopy

**DOI:** 10.1038/s41598-017-09839-1

**Published:** 2017-09-06

**Authors:** Francesco Crisafi, Vikas Kumar, Tullio Scopigno, Marco Marangoni, Giulio Cerullo, Dario Polli

**Affiliations:** 1 IFN-CNR, Dipartimento di Fisica, Politecnico di Milano, Piazza Leonardo da Vinci 32, 20133 Milano, Italy; 2 Dipartimento di Fisica, Università di Roma “La Sapienza”, Roma, I-00161 Italy; 3 Istituto Italiano di Tecnologia, Center for Life Nano Science @Sapienza, Roma, I-00161 Italy; 4 Center for Nano Science and Technology at Polimi, Istituto Italiano di Tecnologia, Milano, 20133 Italy

## Abstract

We introduce a novel configuration for stimulated Raman scattering (SRS) microscopy, called In-line Balanced Detection (IBD), which employs a birefringent plate to generate a time-delayed polarization-multiplexed collinear replica of the probe, acting as a reference. Probe and reference cross the sample at the same position, thus maintaining their balance during image acquisition. IBD can be implemented in any conventional SRS setup, by adding a few simple elements, bringing its sensitivity close to the shot-noise limit even with a noisy laser. We tested IBD with a fiber-format laser system and observed signal-to-noise ratio improvement by up to 30 dB.

## Introduction

Coherent Raman scattering (CRS)^1–3^ is a class of third-order nonlinear microscopy techniques for label-free, non-invasive and non-destructive imaging, with growing applications in biomedical optics and in materials science. In CRS the sample is excited by two synchronized and frequency detuned laser pulses, the pump (ω_p_) and the Stokes (ω_s_). When the pump-Stokes frequency difference matches a vibrational frequency Ω of the molecule, all molecules in the focal volume oscillate in phase, generating a vibrational coherence.

The simplest CRS technique is coherent anti-Stokes Raman scattering (CARS)^4–8^, in which the vibrational coherence is read out by a further interaction with the pump pulse, generating the anti-Stokes frequency ω_as_ = ω_p_ + Ω. The CARS signal is easy to detect, as it is blue-shifted with respect to pump and Stokes; on the other hand, it is overlapped with the background from the third-order non-resonant response of the sample, which distorts and sometimes obscures the resonant signal. Stimulated Raman scattering (SRS)^9–13^ uses the same excitation pulse scheme as CARS, but measures either the pump-induced amplification of the Stokes pulse (the Stimulated Raman Gain, SRG) or the Stokes-induced attenuation of the pump pulse (the stimulated Raman loss, SRL). In both cases, SRS is equivalent to a pump-probe experiment, which detects tiny changes in the transmitted/scattered probe light (the Stokes in the case of SRG or the pump in the case of SRL), of the order of 10^−5^–10^−4^, making it technically more challenging than CARS. SRS almost completely suppresses the non-resonant background and provides a signal that is linear with the concentration of the targeted molecules. For these reasons, SRS is currently the leading CRS microscopy technique for biomedical applications.

CRS microscopy requires rather complex excitation lasers, generating two synchronized picosecond pulse trains with variable frequency detuning. Initial experiments used a picosecond optical parametric oscillator pumped by a bulk solid-state laser^14–17^ that, despite its high output power and low intensity noise, is a complex and expensive system. In recent years, research efforts focused on the development of fiber-format excitation laser systems^18–32^, leveraging their compactness, cost-effectiveness and insensitivity to misalignment. However, while fiber laser systems can be straightforwardly applied to CARS microscopy, the SRS modality has been much more difficult, and in some cases impossible, to be implemented. This is due to the larger amount of intensity noise, even at high Fourier frequencies, that occurs in fiber lasers with respect to their bulk counterparts, caused by the much greater length of the active medium, favoring the build-up of an amplified spontaneous emission background. This excess noise pedestal, which can be as high as 20–30 dB, masks the tiny SRS signal.

For this reason, applications of fiber lasers to SRS microscopy rely on the use of balanced detection schemes, which split off a fraction of the probe beam before the sample and send it to a reference detector, to cancel out the excess laser intensity noise^21, 27, 30, 31, 33^. Such noise suppression, however, turns out to be quite difficult in real microscopy applications, since the spatially dependent transmission and scattering of the sample strongly vary the intensity of the probe beam, bringing it out of balance with the reference beam. To compensate for these variations during image acquisition, several solutions have been proposed. Freudiger *et al*. implemented an auto-balanced detector (ABD), using a sophisticated electronic circuit in which the DC signal levels of the probe and reference detectors are measured and equalized by a variable gain amplifier controlled in a feedback loop^30^. ABD allows maintaining the balance of probe and reference beams during a scan and to increase greatly the signal-to-noise ratio (SNR) of SRS microscopy. An alternative solution, demonstrated by Nose *et al*., is collinear balanced detector (CBD)^33^. In this configuration, a fraction of the probe pulse is split off before the sample, delayed by τ = T_rep_ + Δt, where T_rep_ is the repetition period of the probe laser and Δt is of the order of a few ps, and then collinearly recombined with the probe and sent to the sample. This time-delayed replica of the probe, which is not temporally overlapped with the pump due to the delay Δt, acts as a reference and is detected, together with the probe, by a single photodiode. In the frequency domain, probe and delayed reference show destructive interference at a frequency ω = π/τ ≈ ω_rep_/2, where ω_rep_ = 2π/T_rep_ is the laser repetition rate. This results in effective noise cancellation for pump modulation and lock-in detection at half the laser repetition rate. In the CBD configuration, the probe and reference follow the same pathway and are equally affected by the sample, so that their balance is maintained during acquisition of the image. However, CBD requires a complex optical system (the so-called delay-and-add line) and a delicate alignment procedure for the generation of the reference, which make it difficult to implement experimentally.

In this paper, we propose a novel balanced detection configuration for SRS, which we call in-line balanced detection (IBD), which effectively cancels the noise of the probe beam using a very simple optical setup combined with standard detection electronics. The idea of IBD is to generate the reference as a time-delayed collinear replica of the probe pulse with perpendicular polarization, obtained by passing the probe through a birefringent material of suitable thickness. Probe and reference cross the sample at the same position, thus experiencing equal attenuation due to local absorption/scattering and maintaining their balance during image acquisition. After the sample, they are separated by a polarizing beam splitter and detected with a standard balanced photodiode. IBD can thus be easily implemented in any conventional SRS setup, by the addition of a few simple elements, without requiring sophisticated detector electronics or complex optical delay lines, and bringing its sensitivity close to the shot noise limit even in the presence of a noisy laser source. We tested IBD with a fiber-format laser system and observed a consistent improvement of the SNR up to 30 dB.

## Results and Discussion

### Working principle

**Figure 1:**
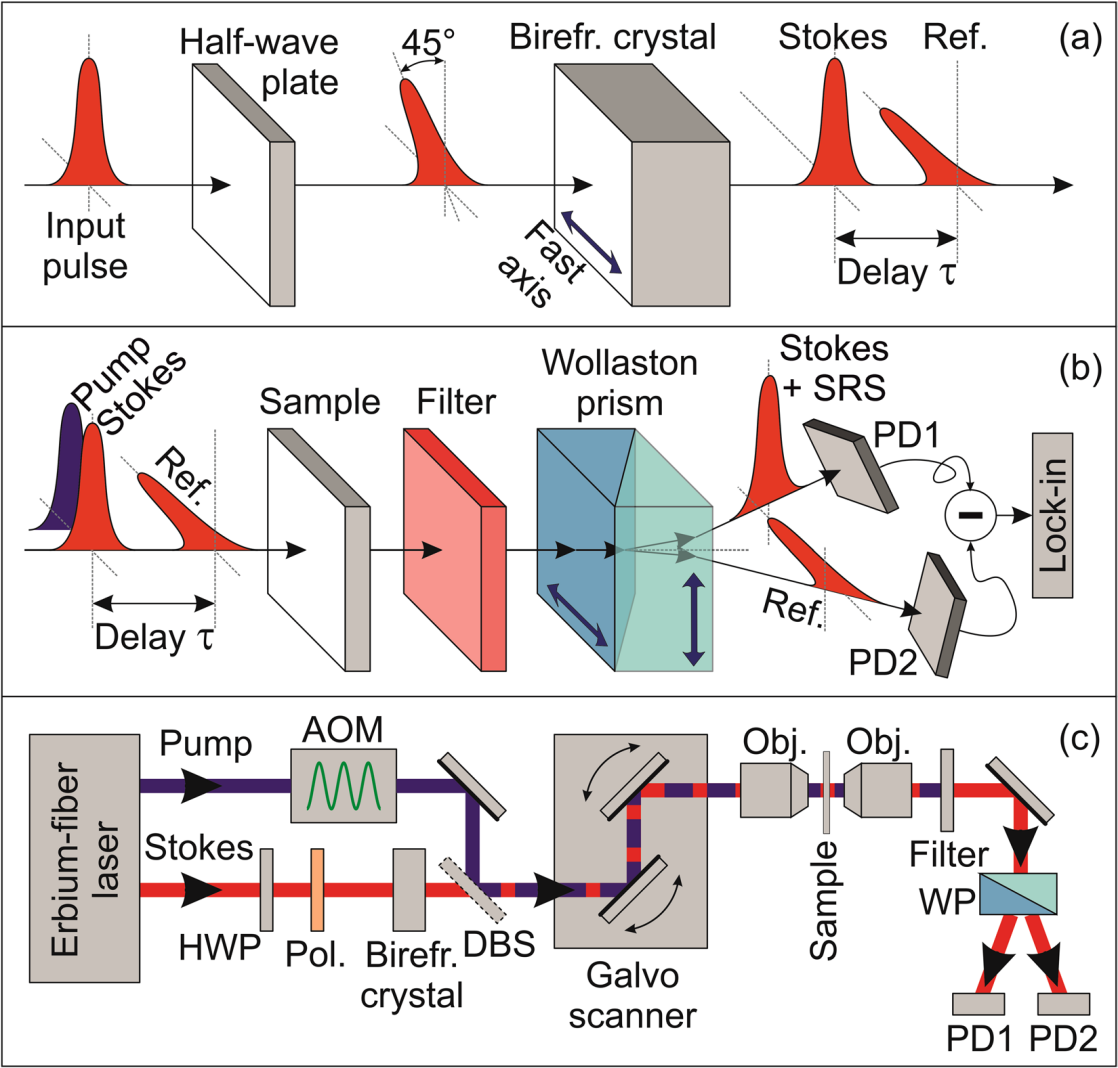
Simple schematic of in-line balanced SRS. (**a**) Generation of the Stokes and reference pulses with perpendicular polarization using a birefringent crystal. (**b**) Interaction of the pulses with the sample and their detection by two photodiodes (PD1, PD2) of a balanced detector. (**c**) Sketch of the entire experimental setup: AOM, acousto-optic modulator; Pol, polarizer; DBS, dichroic beam splitter; Obj, objectives; WP, Wollaston prism.

Figure 1(a) and (b) show the conceptual scheme of IBD-SRS. For the sake of simplicity, we only consider the case of SRG, but a very similar configuration could be applied to SRL. A half-wave plate rotates the polarization of the Stokes pulse, orienting it at 45° with respect to the fast and slow axes of a birefringent crystal. The two pulse projections along the axes propagate with different group velocities ${c}_{0}/{n}_{g}^{o}$ and ${c}_{0}/{n}_{g}^{e}$, where c_0_ is the speed of light in vacuum and ${n}_{g}^{o}({n}_{g}^{e})$ is the ordinary(extraordinary) group index. In Fig. 1(a) the fast axis of the crystal is oriented along the horizontal direction, so that the horizontally polarized replica advances with respect to the vertically polarized one by a delay $\tau =L/{c}_{0}({n}_{g}^{o}-{n}_{g}^{e})$, where L is the crystal length. This delay must be larger than the duration of the pulses and the dephasing time of the vibrational resonances, both typically in the 1–3 ps range. As shown in Fig. 1(b), the trailing pulse is collinearly combined and temporally overlapped with the pump pulse (sharing the same vertical polarization), to serve as the Stokes (probe). The leading pulse, on the contrary, interacts with the unperturbed sample, thus acting as a reference. After the sample, a filter removes the pump radiation and the two replicas are spatially separated by a polarizing beam splitter (such as the Wollaston prism depicted in Fig. 1(b)) and sent to the two photodiodes of a standard balanced detector. A perfect balance of the detected signals is achieved by rotating the half-wave plate. In this way, the output of the balanced detector has a zero DC offset, even for inhomogeneous samples with varying transmission/scattering coefficients, while its AC component at the modulation frequency contains the information on the SRG signal on top of the Stokes pulse, after demodulation with a lock-in amplifier.

Figure 1(c) sketches the experimental setup used for IBD-SRS microscopy. We utilize an Erbium-fiber laser system (for a detailed description see ref. 27) at 40-MHz repetition rate, equipped with two amplified outputs providing 80-fs pulses at ≈1550 nm wavelength with ≈350 mW average power. A periodically poled lithium niobate (PPLN) crystal frequency doubles the output of the first branch, generating ≈2-ps pump pulses at ≈780 nm wavelength with 120-mW average power. The second branch feeds a highly nonlinear fiber generating a supercontinuum, the long-wavelength part of which is frequency doubled in a second PPLN crystal, with a fan-out geometry, generating ≈1-ps Stokes pulses with 2–5 mW average power, continuously tunable in the 930–1060 nm range. In this way, it is possible to vary the pump-Stokes frequency detuning in the 2070–3400 cm^−1^ range, which comprises the entire CH molecular vibrational region (2800–3200 cm^−1^). To ensure a perfect balancing of the photodiodes over the entire Stokes tuning range, we insert a polarizer at 45° after the half-wave plate, because even a zero-order broadband plate presents a non-negligible wavelength dependence of its birefringence and thus of the rotation angle. We chose YVO_4_ as birefringent material because of its high transparency and birefringence $({n}_{g}^{o}-{n}_{g}^{e}\approx 0.2)$, as well as the absence of hygroscopicity. We employed a crystal with length L = 13.3 mm, resulting in a sufficiently long delay of τ ≈ 10 ps between the probe and the reference pulses. We note that the group delay dispersion of YVO_4_ is negligible in our case, as it introduces a variation of τ by ≈300 fs in the 2800–3200 cm^−1^ region, much smaller than the 2-ps pump pulse duration, so that we do not need to actively track the temporal overlap of Stokes and pump while varying their frequency detuning. The pump beam is modulated at 1 MHz by an acousto-optic modulator (AOM), pump and Stokes are collinearly combined by a dichroic beam splitter (DBS) and sent to a home-built laser-scanning microscope (for a detailed description see ref. 32) based on two galvanometric mirrors and a 4-f relay imaging system (not shown in Fig. 1(c)). Stokes and reference beams are detected by a large-area balanced photodiode.

### Noise performances

**Figure 2:**
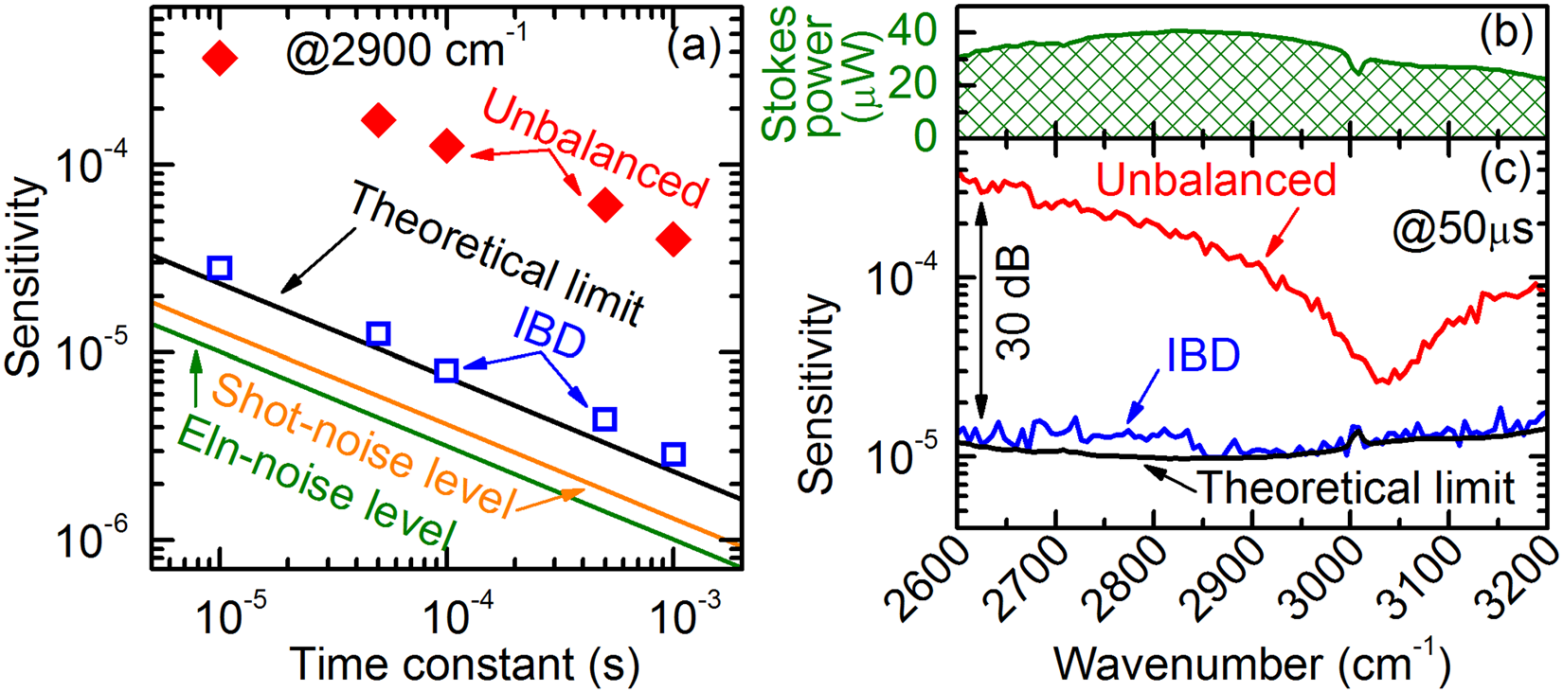
Comparison of the noise performances for unbalanced and in-line balanced detection. (**a**) Measured sensitivity of the detection system at a 2900 cm^−1^ frequency detuning versus time constant (in log-log scale) for single-channel detection (red diamonds) and in-line balanced detection (blue empty squares). Solid lines: sensitivity limits determined by the electronic noise (green), by the shot-noise at each detector (orange) and by the sum of these two contributions (black), which equals the theoretical limit in the adopted experimental conditions. (**b**) Average power of the Stokes pulse train as a function of its frequency detuning with respect to the pump pulse train. (**c**) Sensitivity limits in the C-H spectral region at a 50-μs integration time and at a 1-MHz demodulation frequency for single-channel detection (red) and IBD (blue), together with the theoretical sensitivity limit (black).

We first characterized the performances of our IBD-SRS system and compared them against those of single-channel unbalanced detection. To facilitate the comparison between the two regimes we adopted the same detector and looked at its output in the presence and in the absence of the reference beam. To avoid saturation of the detector in the unbalanced case, we intentionally reduced the incident power to a few tens of µW, as reported by the green curve in Fig. 2(b). Figure 2(a) plots as a function of the measurement time the SRS sensitivity, defined as the minimum detectable SRG signal (with a SNR = 1) and calculated as the ratio between the average root-mean-squared noise voltage at the output of the lock-in amplifier (with no pump) and the DC voltage at the detectors. The sensitivity at 2900 cm^−1^ is shown as red diamonds for the unbalanced case and as blue squares for IBD. In both cases, it follows the expected *t* ^−½^ dependence on the integration time *t*. The laser intensity noise, which is common mode at the two balanced-detector inputs, is effectively canceled out by about 22.6 dB in the case of IBD, thus accelerating the collection process by almost 200 times for a given SNR. Importantly, the residual noise level matches the theoretical limit (black line) given by the sum of the electronic noise from the detector (green line) and of the shot-noise, which is increased by 3 dB with respect to that of a single detector (orange line) due to the presence of the reference channel. When increasing the optical power beyond the 10-µW level here adopted for the sake of comparison, the electronic noise becomes negligible, showing that IBD effectively pushes detection to the shot noise limit.

Figure 2(c) compares the sensitivities of the unbalanced and IBD regimes at various Raman vibrational frequencies and at a fixed 50-μs lock-in time constant. Notably, in our fiber laser system, due to the characteristics of the supercontinuum, the intensity noise of the Stokes pulses is wavelength dependent, with a sharp increase at the edges of the tuning range. Nonetheless, the sensitivity measured with IBD corresponds to the theoretical limit over the whole spectrum, thus attesting nearly shot-noise limited performance at any frequency detuning. We note that, with a more powerful Stokes beam (with 1–10 mW power level at the detector, still below the damage threshold of biological samples), the shot-noise limit would be reduced by approx. one order of magnitude, thus improving the quality of the collected images with IBD technique and/or reducing the required integration time accordingly.

### In-line balanced detection SRS spectroscopy and microscopy

**Figure 3:**
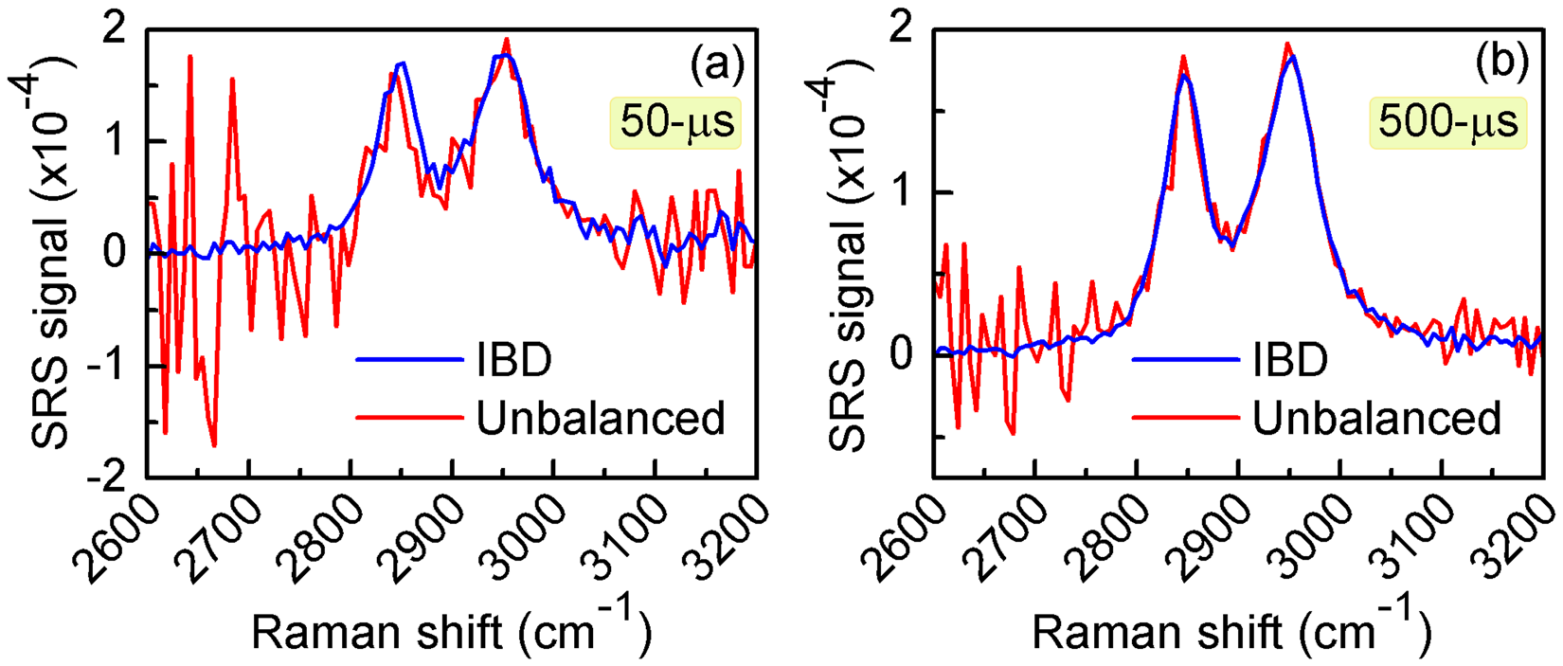
SRS spectra of Methanol solution. Comparison of SRS spectra for IBD (blue) and unbalanced (red) schemes at integration times of 50-μs (**a**) and 500-μs (**b**).

To demonstrate the effectiveness of the IBD scheme in an SRS configuration, we first acquired SRG spectra of pure methanol. The results are plotted in Fig. 3(a) and (b) for 50-μs and 500-μs lock-in time constants, respectively, both for IBD-SRS (blue curves) and for unbalanced SRS (red curves) under the same experimental conditions. The SRS spectrum of methanol is in good agreement with the spontaneous Raman spectrum, displaying two peaks at 2840 and 2951 cm^−1^, corresponding to the symmetric and antisymmetric CH_3_ stretching modes, respectively. The IBD-SRS spectra are far cleaner than the unbalanced SRS spectra at both integration times.

**Figure 4:**
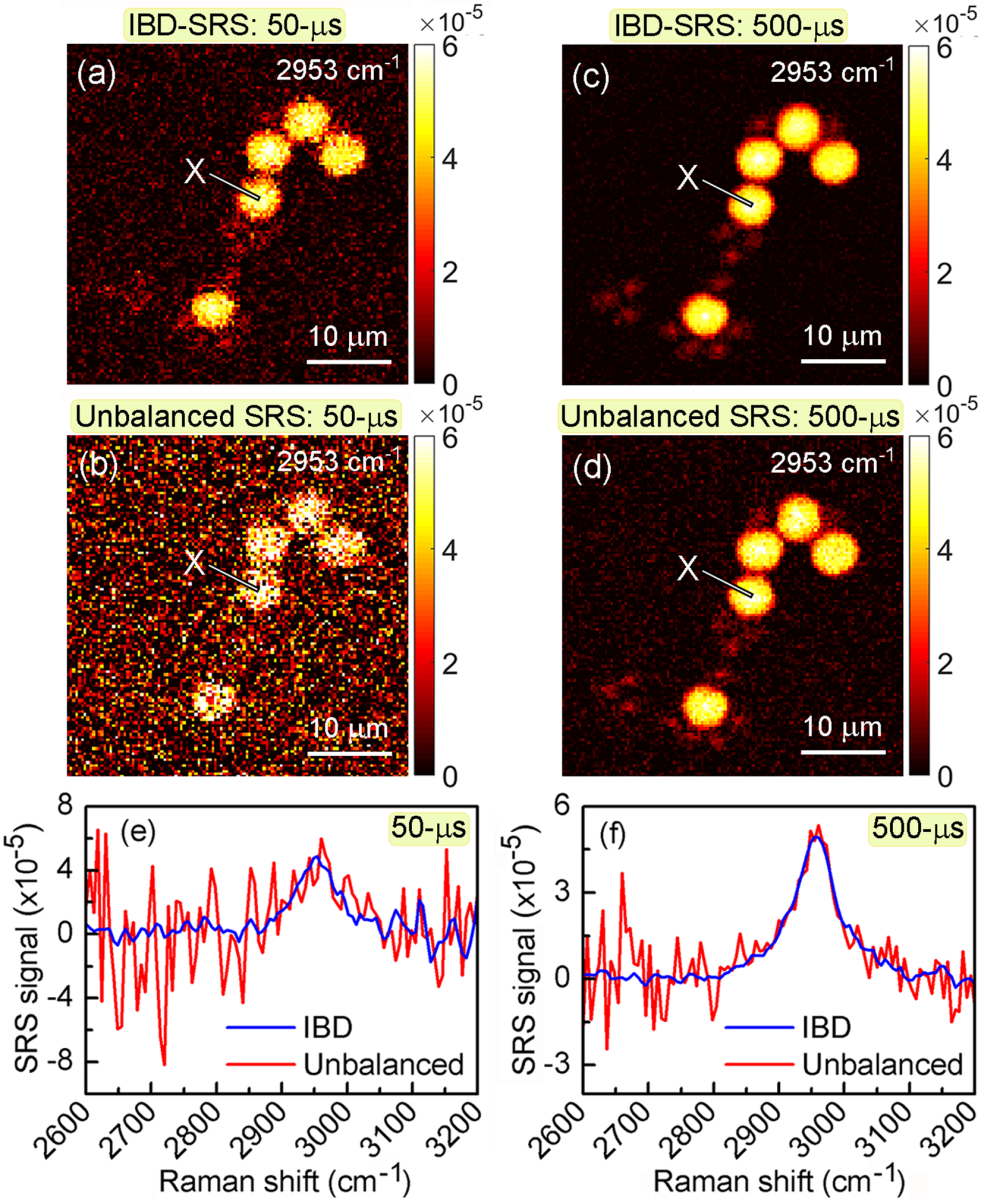
Comparison between unbalanced and IBD-SRS imaging. (**a**–**d**) 120 × 120 pixels images of a 40 × 40 μm^2^ sample area of a blend of 6-μm PMMA and 3-μm PS beads acquired at the PMMA Raman resonance of 2953 cm^−1^. (**a**,**c**) IBD-SRS images at integration times of 50-μs and 500-μs, respectively. (**b**,**d**) as above, but under unbalanced SRS as a sake of comparison. (**e**,**f**) SRS spectra of PMMA at the point X indicated in the panels above, under IBD- (blue) and unbalanced-SRS (red) at integration times of 50-μs and 500-μs, respectively.

As a further step, we tested the imaging capabilities of IBD-SRS in laser scanning microscopy. First, we imaged a mixture of 6-μm poly-methyl methacrylate (PMMA) and 3-μm polystyrene (PS) beads dispersed on a glass substrate. Figure 4 shows images at the PMMA resonance (2953 cm^−1^) for IBD-SRS (Fig. 4(a–c)) and unbalanced SRS (Fig. 4(b–d)) at two different pixel dwell times: 50-μs (panels a-b) and 500-μs (panels c-d). In both cases, the IBD-SRS images show superior quality with respect to the corresponding unbalanced SRS images. The SRG spectra collected at point X of the PMMA bead (as indicated in the images) in the IBD-SRS (in blue) and the unbalanced SRS (in red) schemes are reported in Fig. 4(e) for 50-μs and Fig. 4(f) for 500-μs integration times. Again, while the noise in the case of unbalanced SRS is strongly wavelength dependent, due to the varying stability of the Stokes pulse, it is much lower and nearly wavelength independent for IBD-SRS. We note that the two small side-peaks of the 3000 cm^−1^ CH_3_ stretching are not visible with our spectral resolution (≈30 cm^−1^, set by the bandwidth of the Stokes pulse) which could be easily improved by employing a longer PPLN crystal for spectral compression^19, 34^.

**Figure 5:**
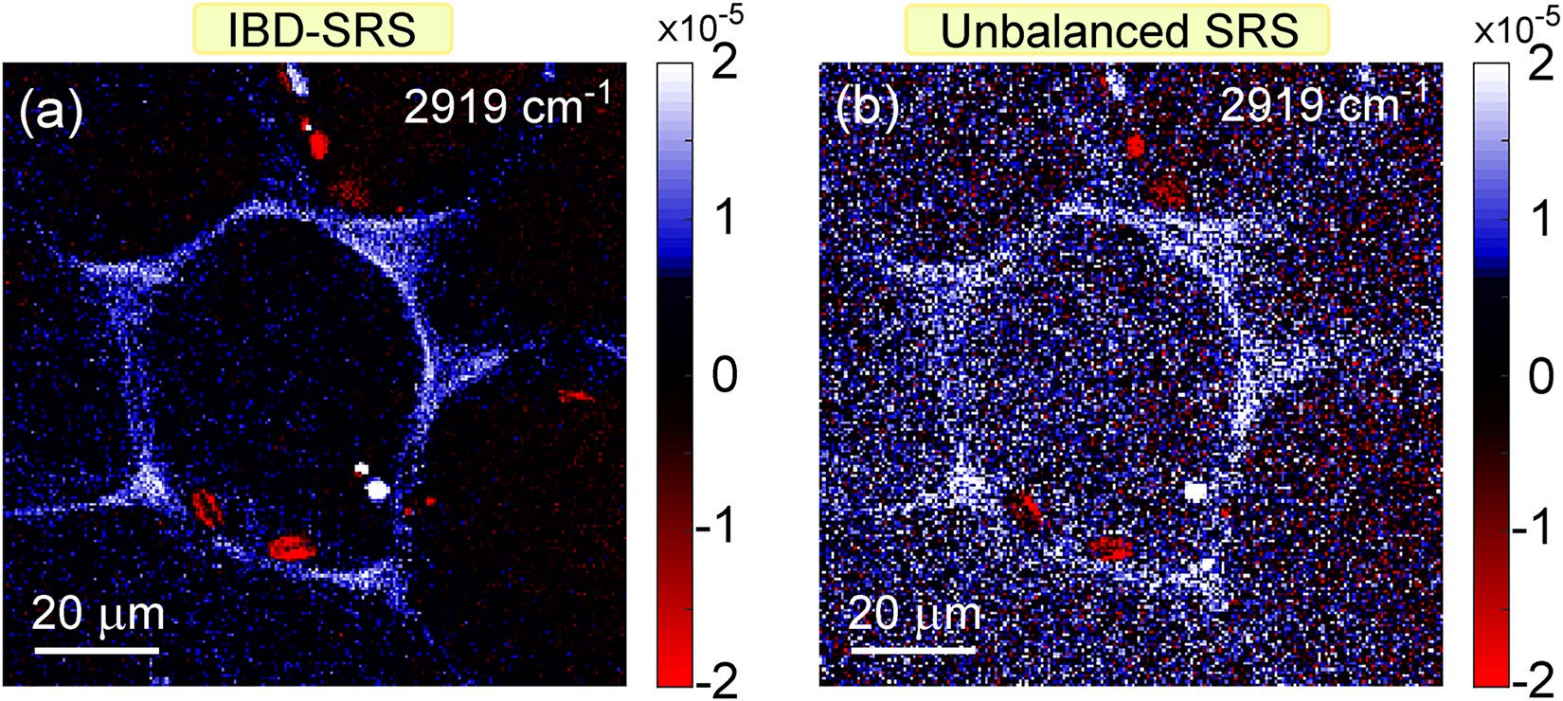
Comparison of IBD and unbalanced SRS for biological imaging. 200 × 200 pixels images of a 100 × 100 μm^2^ sample area of cells in cross-section of a leaf petiole of an *Elodea* plant acquired at 2919 cm^−1^ with 1-ms integration time. (**a**) IBD-SRS. (**b**) Unbalanced SRS.

Figure 5 shows SRS images of a thin cross-section of the leaf petiole from a fresh *Elodea* aquatic plant, sandwiched with water between two glass slides. Images were acquired at 2919 cm^−1^ vibrational frequency, tuned to the typical response of the cell walls that are rich in lignin and cellulose. The images confirm the superior SNR of IBD-SRS (Fig. 5(a)) over the single-channel unbalanced SRS case (Fig. 5(b)) also for biological samples with much higher optical inhomogeneity.

**Figure 6:**
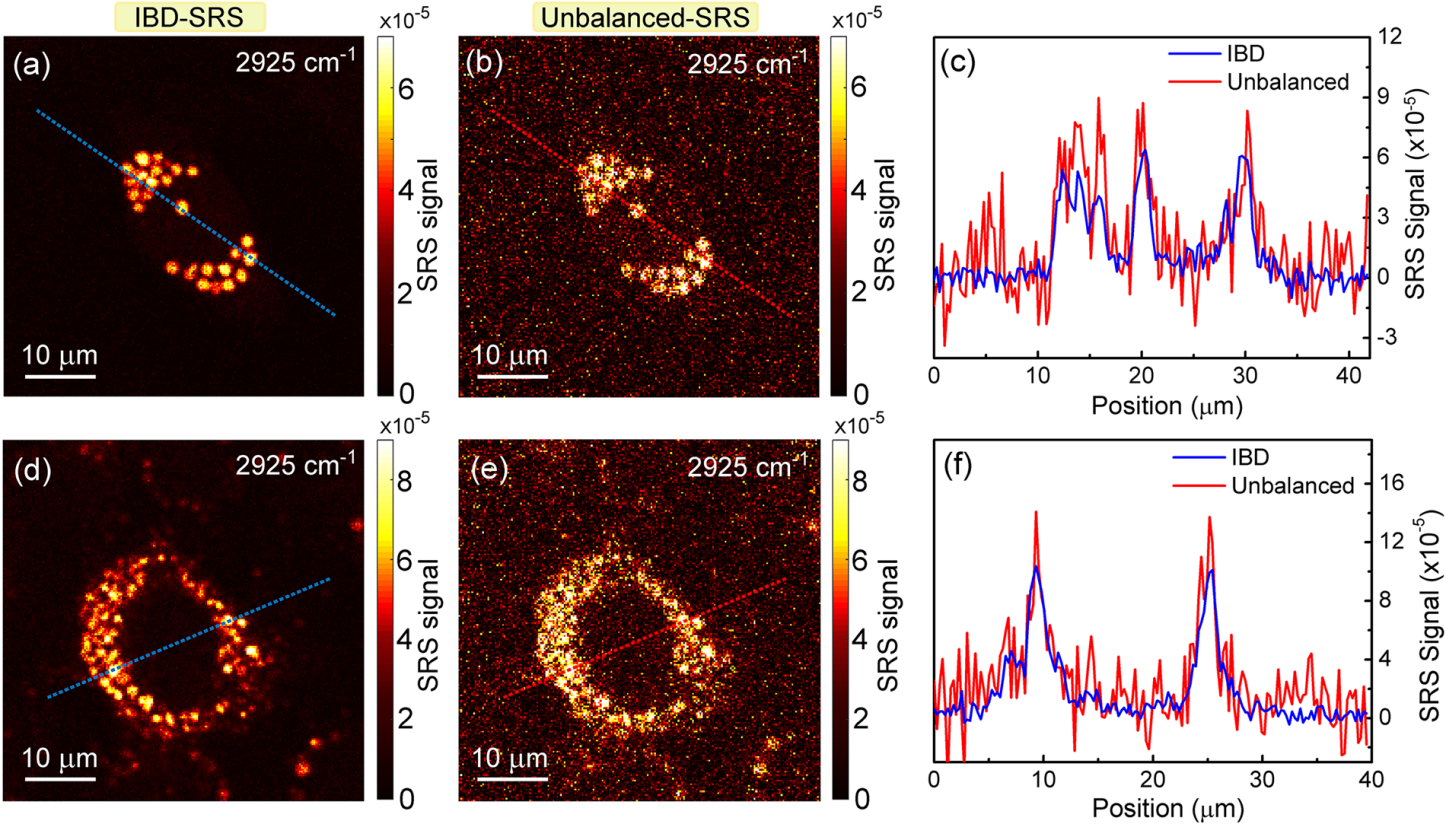
Imaging of liver cells. Comparison of IBD (**a**,**d**) with unbalanced (**b**,**e**) SRS imaging for HepaRG (**a**–**c**) and HuH7 (**d**–**f**) liver cells treated with oleic acid. (**c**,**f**) Plot of the SRS signals along the two cross sections indicated in the images. Integration time: 500 µs per pixel.

**Figure 7:**
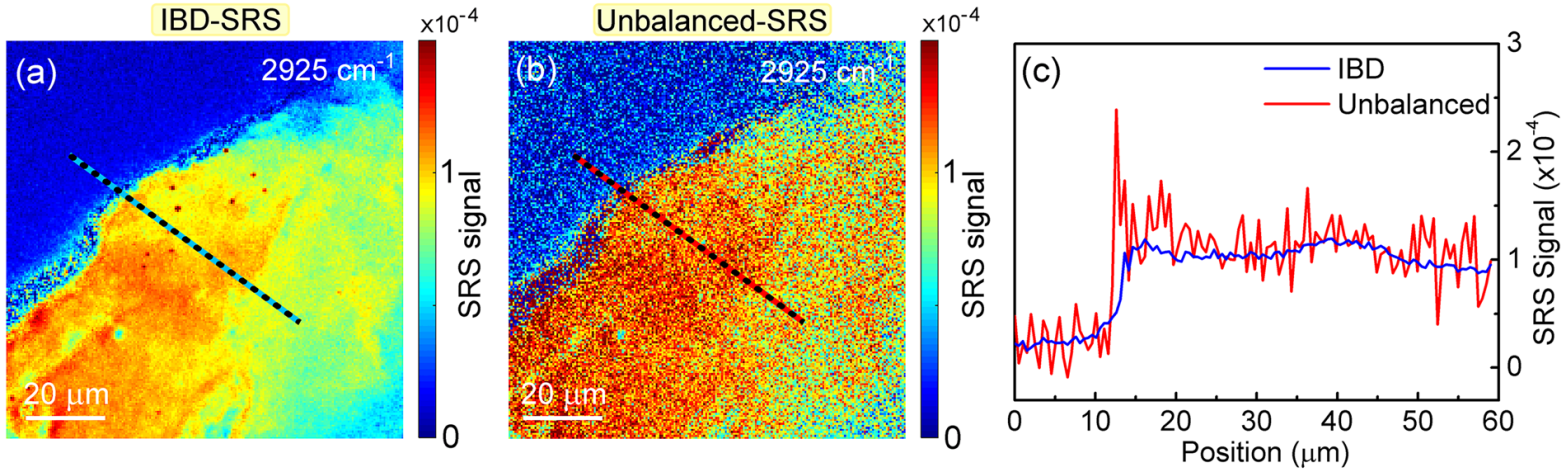
Imaging of a thick tissue from a bovine liver. Comparison of IBD (**a**) and unbalanced (**b**) SRS imaging. (**c**) Plot of the SRS signals along the cross sections indicated in the images. Integration time: 500 µs per pixel.

As a final step, we imaged liver cells (Fig. 6) and tissue (Fig. 7). We analyzed two cell lines^35, 36, 37^: HuH7 (Fig. 6(a–c)), a well differentiated hepatocyte derived human carcinoma cell line, and HepaRG cells (Fig. 6(d–f)), derived from a human bipotent hepatic progenitor cell line, capable to differentiate toward two different cell phenotypes. Cells were prepared as described in the Supplementary Methods section online. We could easily detect lipid droplets, as shown in Fig. 6, showing the SRS signal at 2925 cm^−1^, tuned at the peak of CH stretching. SNR for the IBD case is 20 for the HepaRG cells (Fig. 6(a)) and 23 for the HuH7 cells (Fig. 6(d)). SNR for the unbalanced cases are 4.5 (Fig. 6(b)) and 6 (Fig. 6(e)) for the two cells, respectively. In panels (c) and (f) we plot two horizontal cuts along the dashed lines, to highlight the better signal-to-noise ratio of IBD with respect to the unbalanced case. We also performed a three-dimensional scan of the cells by acquiring a series of x-y images at various z depths across the cells. This allowed us to reconstruct the volumetric distribution of the lipid droplets, as shown in Supplementary Movies S1 and S2.

Imaging the liver cells was propaedeutic for assessing the performances of our IBD technique in performing SRS microscopy of a thick sample such as a liver tissue slice. This is of paramount importance because it enables us to verify if the light polarization is preserved during propagation in a tissue, a prerequisite for the proper functioning of IBD. In fact, if the two perpendicularly polarized Stokes replicas (one for the signal, one for the reference) rotated or lost polarization, some SRS signal would not anymore be detected on the “+” channel of the photodiode but conversely be detected on the “−” channel, so that after subtraction one would obtain a lower SRS signal. Therefore, we imaged a fresh bovine liver tissue with ≈30-µm thickness (the maximum obtainable with our cryotome for histopathology) at various depths with 3-µm steps, slightly larger than our spatial resolution in the axial direction (≈2 µm). The complete set of SRS images are reported in Supplementary Figures S3 and S4. In Fig. 7 we plot the x-y cross-sectional image of the SRS signal at 2925 cm^−1^, tuned at the peak of the lipid signal, measured in the middle axial position of the tissue, for both unbalanced (Fig. 7(b)) and IBD (Fig. 7(a)) cases. Figure 7(c) shows a cross section of the SRS images along the dashed line. The SRS signal for the IBD case (blue line) is clearly less noisy and it nicely follows the SRS signal of the unbalanced case (red line), though with a <5% reduction of the signal. This is suggestive of a minor depolarization of the Stokes beam in propagating though the 30-µm-thick tissue. By extrapolating this result to thicker tissues, one could foresee that our IBD technique should be applicable to tissues with thicknesses up to 100–200 µm, preserving a significant portion of the SRS signal. We note that this depolarization effect, if present, would equally affect the capability of the instrument in imaging both the lower and the upper portions of the tissue, as sample anisotropy/birefringence is a linear effect.

## Summary

In conclusion, we have presented a very simple scheme for balanced detection in SRS microscopy. Our method uses a birefringent crystal inserted in the probe beam pathway to generate a time-advanced and collinear reference pulse, which is separated from the probe after the sample by a polarizing beam splitter. Since probe and reference impinge on the same position of the sample, they experience the same absorption/scattering losses and thus remain balanced even in the presence of inhomogeneous samples. With respect to previously proposed approaches for balanced detection in microscopy, our scheme has the advantages of simplicity and of requiring standard detectors. Using a few simple off-the-shelf components, it can be used to upgrade any existing SRS microscopy setup, bringing its performances close to the shot-noise limit. This is especially important with fiber lasers, which trade off low cost and compactness with an increased intensity noise. The combination of fiber lasers with IBD could lead to CRS microscopy systems with greatly simplified architecture, fostering their application in a wide class of problems in biophotonics.

## Electronic supplementary material

Supplementary Information

Supplementary Movies S1

Supplementary Movies S2

## References

[1] Camp CH, Cicerone MT (2015). Chemically sensitive bioimaging with coherent Raman scattering. Nat. Photonics.

[2] Cheng JX, Xie XS (2015). Vibrational spectroscopic imaging of living systems: An emerging platform for biology and medicine. Science.

[3] Orringer DA (2017). Rapid intraoperative histology of unprocessed surgical specimens via fibre-laser-based stimulated Raman scattering microscopy. Nat. Biomed. Eng..

[4] Zumbusch A, Holtom GR, Xie XS (1999). Three-Dimensional Vibrational Imaging by Coherent Anti-Stokes Raman Scattering. Phys. Rev. Lett..

[5] Hashimoto M, Araki T, Kawata S (2000). Molecular vibration imaging in the fingerprint region by use of coherent anti-Stokes Raman scattering microscopy with a collinear configuration. Opt. Lett..

[6] Evans CL, Xie XS (2008). Coherent Anti-Stokes Raman Scattering Microscopy: Chemical Imaging for Biology and Medicine. Annu. Rev. Anal. Chem..

[7] Camp CH (2014). High-speed coherent Raman fingerprint imaging of biological tissues. Nat. Photonics.

[8] Evans CL (2005). Chemical imaging of tissue *in vivo* with video-rate coherent anti-Stokes Raman scattering microscopy. Proc Natl Acad Sci USA.

[9] Freudiger CW (2008). Label-Free Biomedical Imaging with High Sensitivity by Stimulated Raman Scattering Microscopy. Science.

[10] Nandakumar P, Kovalev A, Volkmer A (2009). Vibrational imaging based on stimulated Raman scattering microscopy. New J. Phys..

[11] Saar BG (2010). Video-Rate Molecular Imaging *in Vivo* with Stimulated Raman Scattering. Science.

[12] Ozeki Y (2012). High-speed molecular spectral imaging of tissue with stimulated Raman scattering. Nat. Photonics.

[13] Fu D (2012). Quantitative Chemical Imaging with Multiplex Stimulated Raman Scattering Microscopy. J. Am. Chem. Soc..

[14] Ganikhanov F (2006). Broadly tunable dual-wavelength light source for coherent anti-Stokes Raman scattering microscopy. Opt. Lett..

[15] Jurna M, Korterik JP, Otto C, Herek JL, Offerhaus HL (2009). Vibrational Phase Contrast Microscopy by Use of Coherent Anti-Stokes Raman Scattering. Phys. Rev. Lett..

[16] Downes A, Mouras R, Elfick A (2009). A versatile CARS microscope for biological imaging. J. Raman Spectrosc..

[17] Wang Z, Zheng W, Hsu C-YS, Huang Z (2016). Polarization-resolved hyperspectral stimulated Raman scattering microscopy for label-free biomolecular imaging of the tooth. Appl. Phys. Lett..

[18] Tauser F, Leitenstorfer A, Zinth W (2003). Amplified femtosecond pulses from an Er:fiber system: Nonlinear pulse shortening and selfreferencing detection of the carrier-envelope phase evolution. Opt. Express.

[19] Marangoni M (2009). Fiber-format CARS spectroscopy by spectral compression of femtosecond pulses from a single laser oscillator. Opt. Lett..

[20] Krauss G (2009). Compact coherent anti-Stokes Raman scattering microscope based on a picosecond two-color Er: fiber laser system. Opt. Lett..

[21] Gambetta A (2010). Fiber-format stimulated-Raman-scattering microscopy from a single laser oscillator. Opt. Lett..

[22] Andresen ER, Berto P, Rigneault H (2011). Stimulated Raman scattering microscopy by spectral focusing and fiber-generated soliton as Stokes pulse. Opt. Lett..

[23] Baumgartl M (2012). All-fiber laser source for CARS microscopy based on fiber optical parametric frequency conversion. Opt. Express.

[24] Gottschall T (2012). Fiber-based source for multiplex-CARS microscopy based on degenerate four-wave mixing. Opt. Express.

[25] Chemnitz M (2012). Widely tuneable fiber optical parametric amplifier for coherent anti-Stokes Raman scattering microscopy. Opt. Express.

[26] Lefrancois S (2012). Fiber four-wave mixing source for coherent anti-Stokes Raman scattering microscopy. Opt. Lett..

[27] Kumar V (2012). Coherent Raman spectroscopy with a fiber-format femtosecond oscillator. J. Raman Spectrosc..

[28] Lamb ES (2013). Fiber optical parametric oscillator for coherent anti-Stokes Raman scattering microscopy. Opt. Lett..

[29] Coluccelli N (2014). Er/Tm: fiber laser system for coherent Raman microscopy. Opt. Lett..

[30] Freudiger CW (2014). Stimulated Raman scattering microscopy with a robust fibre laser source. Nat. Photonics.

[31] Riek C (2016). Stimulated Raman scattering microscopy by Nyquist modulation of a two-branch ultrafast fiber source. Opt. Lett..

[32] Crisafi F (2018). Multimodal nonlinear microscope based on a compact fiber-format laser source. Spectrochimica Acta Part A: Molecular and Biomolecular Spectroscopy.

[33] Nose K (2012). Sensitivity enhancement of fiber-laser-based stimulated Raman scattering microscopy by collinear balanced detection technique. Opt. Express.

[34] Marangoni M (2007). Narrow-bandwidth picosecond pulses by spectral compression of femtosecond pulses in a second-order nonlinear crystal. Opt. Express.

[35] Guguen-Guillouzo, C. & Guillouzo, A. General Review on *In Vitro* Hepatocyte Models and Their Applications in *Hepatocytes*. *Methods in Molecular Biology (Methods and Protocols)* vol. 640 (ed. Maurel, P.) 1–40 (Humana Press, 2010). 10.1007/978-1-60761-688-7_1 20645044

[36] Guillouzo A (2007). The human hepatoma HepaRG cells: A highly differentiated model for studies of liver metabolism and toxicity of xenobiotics. Chem.-Biol. Interact..

[37] Nunn, A. D. G. *et al*., The histone deacetylase inhibiting drug Entinostat induces lipid accumulation in differentiated HepaRG cells. *Sci. Rep.* **6**, 28025 (2016). 10.1038/srep28025 PMC4913258 27320682

