# Supplementary material for: In-line balanced detection stimulated Raman scattering microscopy
Source: Sci Rep. 2017 Sep 6;7:10745. doi: 10.1038/s41598-017-09839-1 (PMC5587718; doi:10.1038/s41598-017-09839-1)
Supplement: Supplementary file 1 — Supplementary Information [file 41598_2017_9839_MOESM1_ESM.pdf]

In-line balanced detection stimulated Raman scattering microscopy

Francesco Crisafi, Vikas Kumar, Tullio Scopigno, Marco Marangoni, Giulio Cerullo, Dario Polli

Supplementary Methods: cell preparation.

Human hepatoma HepaRG cells were seeded at a density of 2.6×10^4 cells/cm^2 in William's E medium with GlutamaX (GiBco) supplemented with 10% fetal bovine serum, 100 U/mL penicillin, 100 µg/mL streptomycin, 5 µg/mL insulin and 50 µM hydrocortisone hemi-succinate. In one week cells reached confluence with a doubling time of around 24 h and were shifted to the same medium supplemented with 2% dimethyl sulfoxide for a further two weeks to obtain confluent differentiated cultures. Huh7 cells were maintained in Dulbecco's Modified Eagle Medium supplemented with 10% fetal bovine serum, 1% penicillin/streptomycine and 1% glutamine. After differentiation of HepaRG cells, HepaRG and HuH7 cells were incubated for 1 day with a solution of sodium oleate (250 µM). After the period of incubation, the cells were fixed with 4% formaldehyde ready for imaging.

Supplementary Figures

In the following figures we plot a series of 11 images collected in a bovine liver tissue at various depths, with 3-µm step in the axial direction. The SRS signal has been collected at 2925 cm^{-1}, tuned at the peak of the lipid signal.

The first figure has been plotted with a fixed color-scale for the SRS signal, while the second has been plotted re-scaling the SRS signal intensity for each image to use the full colorscale available from the blue to the red. Different structures can be seen at different depths.

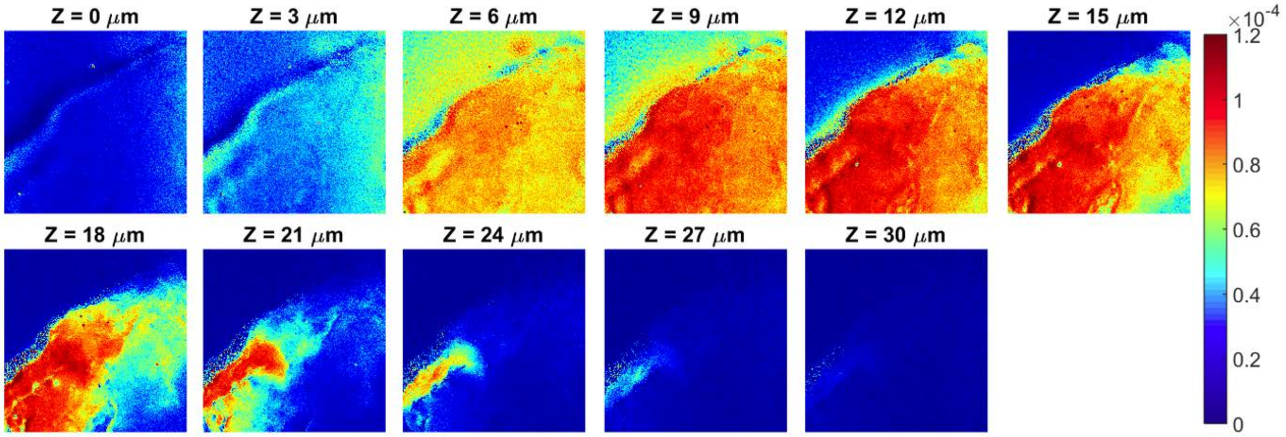

Figure S3. Images of a bovine liver tissue at different depths as indicated. Image size: 100x100 µm^2.

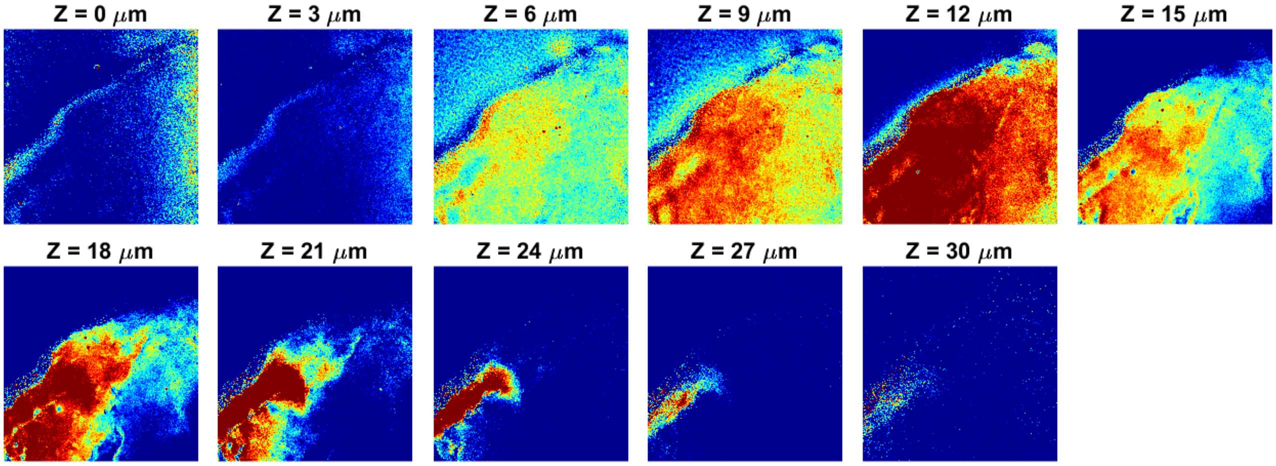

Figure S4. Images of a bovine liver tissue at different depths as indicated. Image size: 100x100 μm^2.
